# Unravelling Tinengotinib’s Mechanistic Landscape in Triple-Negative Breast Cancer Via Network Pharmacology and in Silico Simulation Techniques

**DOI:** 10.1007/s12013-025-01907-y

**Published:** 2025-10-16

**Authors:** Musab Ali, Narasimham L. Parinandi, Archimede Rotondo, Giuseppe Pellicane, Shahzeb Khan, Mahmoud E. S. Soliman

**Affiliations:** 1 Molecular Bio-Computation and Drug Design Research Group, School of Health Sciences, University of KwaZulu Natal, Westville Campus, Durban, 4001 South Africa; 2 Division of Pulmonary, Critical Care and Sleep Medicine Department of Medicine, Davis Heart and Lung Research Institute, Weber Medical Center, The Ohio State University, Columbus, OH USA; 3 Dipartimento di Scienze Biomediche, Odontoiatriche e delle Immagini Morfologiche e Funzionali, Università degli Studi di Messina, Messina, I-98125 Italy; 4 School of Chemistry and Physics, University of Kwazulu-Natal and National Institute for Theoretical and Computational Sciences (NITheCS), Pietermaritzburg, 3209 South Africa; 5 Institute of Health and Social Care, School of Pharmacy, Optometry and Medical Sciences, University of Bradford, BD7 1DP, West Yorkshire, Bradford, UK

**Keywords:** Triple-negative breast cancer, Network pharmacology, Tinengotinib, Molecular docking, Molecular dynamics simulation

## Abstract

Triple-negative breast cancer (TNBC) is a highly aggressive and heterogeneous subtype with limited treatment options and poor clinical outcomes. Tinengotinib (TT-00420), a spectrum-selective multi-kinase inhibitor, has emerged as a promising candidate for targeting multiple dysregulated pathways in TNBC. This study employed an integrative in silico strategy—combining network pharmacology, molecular docking, molecular dynamics (MD) simulations, and binding free energy (MMGBSA) calculations—to investigate the therapeutic potential of tinengotinib. Network pharmacology revealed that tinengotinib modulates key TNBC-associated pathways, including PI3K-Akt, ErbB, and focal adhesion, which are central to proliferation, apoptosis, and therapy resistance. Molecular docking demonstrated strong binding affinities with hub proteins, particularly PTK2 (-10.7 kcal/mol), MAPK8 (-10.6 kcal/mol), EGFR (-9.5 kcal/mol), and ESR1 (-9.4 kcal/mol). MD simulations confirmed stable binding and favorable conformational dynamics across all complexes, with EGFR and PTK2 showing reduced solvent accessibility indicative of structural compaction, while ESR1, SRC, and MAPK8 displayed increased solvent exposure. MMGBSA analysis further validated the thermodynamic stability of these interactions, with PTK2 and MAPK8 exhibiting the most favorable binding free energies. Collectively, these results highlight tinengotinib’s multitarget mechanism through stable interactions with key oncogenic proteins, providing a strong molecular rationale for its continued clinical development in TNBC management.

**Supplementary Information:**

The online version contains supplementary material available at 10.1007/s12013-025-01907-y.

## Introduction

Breast cancer (BC) is the most frequently diagnosed malignancy in women and stands as the second-highest contributor to cancer-related deaths globally [1, 2]. According to the GLOBOCAN 2020 database, there were approximately 2.3 million new breast cancer cases and 685,000 deaths globally, accounting for 11.7% of all cancer cases and 6.9% of cancer deaths [3]. Breast cancer is characterized by significant heterogeneity, manifesting in a wide range of histopathological subtypes, variable natural progression, distinct clinical behaviors, and differential responses to therapeutic interventions [4, 5]. Based on the expression profiles of molecular markers—namely estrogen receptor (ER), progesterone receptor (PR), and human epidermal growth factor receptor 2 (HER2)—breast cancer is classified into three principal subtypes: hormone receptor (HR)-positive, HER2-positive, and triple-negative breast cancer (TNBC) [6, 7].

TNBC embodies a distinct subtype of breast cancer defined by the absence of hormone receptor expression and the lack of HER2 gene amplification [8–11]. It was originally conceptualized as a distinct clinical entity, closely aligned with the basal-like subtype that emerged from pioneering gene expression microarray studies in the early 2000s [12]. TNBC represents approximately 15% to 20% of all breast carcinomas [13], and it is more prevalent in premenopausal women, African American women, and carriers of deleterious breast cancer susceptibility gene 1 and 2 (BRCA 1/2) mutation [14]. Compared to regular types, TNBC is associated with a poorer prognosis with patients often presenting with more aggressive disease characteristics [14–16], increased likelihood of early relapse [17, 18], and a reduction in overall survival [19, 20].

In essence, triple-negative breast cancer (TNBC) is a umbrella term encompassing a diverse spectrum of entities that exhibit distinct biological characteristics and clinical behaviors, with significant variations at the genetic, transcriptional, histological, and clinical levels [21].

Owing to distinct molecular phenotypes, TNBC lacks sensitivity to endocrine therapy and molecularly targeted treatments. Consequently, chemotherapy remains the primary systemic therapeutic approach. However, the effectiveness of conventional postoperative adjuvant chemoradiotherapy is limited, often failing to achieve sustained disease control. Aggressive clinical nature, poor prognosis and lack of therapeutic agents [22, 23], placed TNBC as an interesting and challenging topic in breast cancer research.

Network pharmacology (NP) represents a paradigm shift in the field of drug discovery by moving away from the traditional “one drug–one target” approach towards a systems-level understanding of biological interactions. This discipline integrates systems biology, bioinformatics, and network theory data to construct comprehensive interaction maps connecting drugs, their molecular targets, and the complex networks governing disease pathogenesis [24]. One of the key strengths of network pharmacology is its ability to capture the inherent complexity and redundancy of biological systems. By mapping protein-protein interactions, gene regulatory circuits, and metabolic pathways, researchers can identify critical network hubs and bottlenecks that may serve as more effective therapeutic targets. This systems-based approach is particularly important in multifactorial diseases such as cancer, where dysregulation occurs across multiple signalling pathways and a single-target strategy often falls short [24, 25]. NP is widely applied in several key areas of drug discovery. Through the construction of drug-target networks, scientists can pinpoint central nodes within biological pathways that are essential for disease progression, thus enabling identification of novel targets that might be missed when considering single molecules in isolation [26]. Additionally, NP allows for the systematic exploration of existing drugs to uncover previously unrecognized therapeutic potentials, hence facilitating drug repurposing [27]. Most importantly, NP provides a rational framework for the design of multi-target drugs that can achieve synergistic therapeutic effects, reduce the likelihood of resistance, and improve clinical outcomes [27]. The integration of omics data has further empowered network pharmacology by offering comprehensive datasets that enhance the fidelity of interaction maps and ease the discovery of novel biomarkers and therapeutic targets [25–27].

Tinengotinib (TT-00420) is an orally administered, spectrum‐selective multi‐kinase inhibitor that has garnered significant attention in preclinical and early clinical studies for its activity against triple‐negative breast cancer (TNBC). Designed to target multiple key-signalling pathways, tinengotinib inhibits critical kinases involved in cell proliferation, angiogenesis, and immune regulation [28]. Preclinical studies have demonstrated that tinengotinib exhibits robust antiproliferative effects across various TNBC cell lines in vitro while sparing luminal breast cancer cells. This selective inhibition not only underscores its potential utility in treating TNBC but also points to its ability to modulate tumor-specific signalling networks [28]. The clinical potential of tinengotinib is further supported by early-phase clinical data. A recent first-in-human phase I study reported that tinengotinib was well tolerated, with manageable dose-limiting toxicities [29]. While extensive experimental studies have established its efficacy in inhibiting tumor growth, the detailed molecular interactions and binding dynamics between tinengotinib and its various targets are yet to be characterized.

**Fig. 1:**
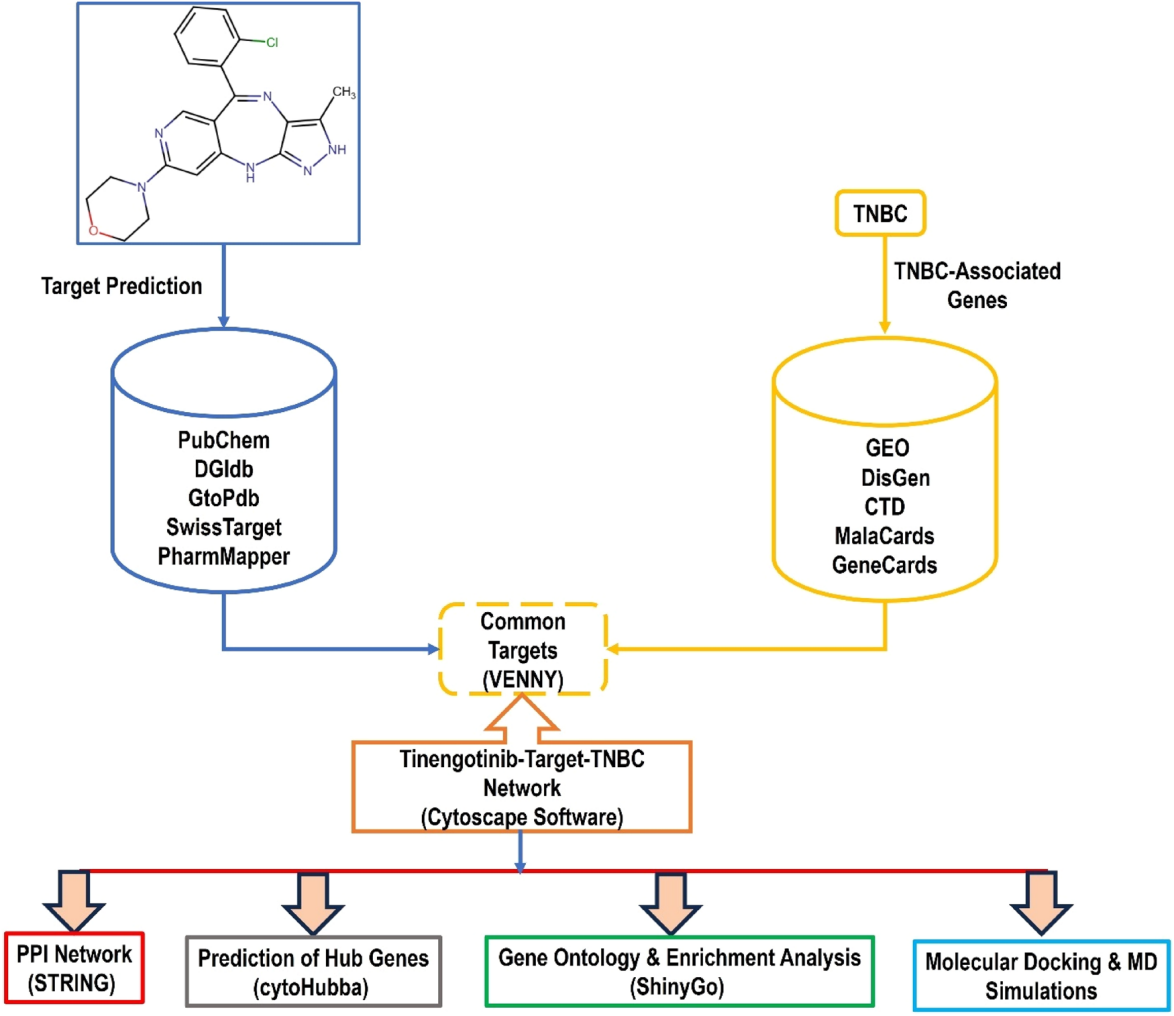
A schematic flow chart for steps used in this study

The present work aims to bridge this knowledge gap by employing a combined network analysis, molecular docking, and molecular dynamics simulation strategy to systematically decipher the interactome of tinengotinib in TNBC. Through integrative methodology (Fig. 1), we intend to spot key protein targets and their interconnections within the TNBC signalling network and elucidate the binding affinities and molecular interactions governing tinengotinib’s activity.

We speculate that our study would help formulate a deeper understanding of the molecular basis underlying tinengotinib’s multi-target effects; not only decipher its clinical potential in TNBC but also contribute to the broader field of cancer therapy.

## Methodology

### Genetic Mapping of TNBC

TNBC-associated genes were collectively mapped from several databases to ensure comprehensive coverage of all possible genes. These include Gene Expression Omnibus (GEO) (https://www.ncbi.nlm.nih.gov/geo/) [30], DISGENET (https://disgenet.com/) [31], MalaCards (https://www.malacards.org/) [32], GeneCards (https://www.genecards.org/) [33], and Comparative Toxicogenomics Database (https://ctdbase.org/) [34].

The GSE dataset “GSE27447” was selected from the GEO database and assessment of differentially expressed genes (DEG) were conducted using the built-in GEO2R tool. The 19 samples of the dataset were categorized into Control (non-TNBC) and Test (TNBC), respectively. Analysis parameters included a significance level (P value < 0.05), fold change threshold (log2FC > 2) and limma precision weights. For the rest of the databases, only search results under the query “Triple-Negative Breast Cancer” were explicitly collected.

### Targets Prediction of Tinengotinib

Similarly, protein targets of tinengotinib were mapped from PubChem (https://pubchem.ncbi.nlm.nih.gov/) [35], Drug–Gene Interaction Database (DGIdb) (https://dgidb.org/) [36], Guide to Pharmacology (GtoP) (https://www.guidetopharmacology.org/) [37], Swiss Target Prediction (http://www.swisstargetprediction.ch/) [38], and PharmMapper (https://www.lilab-ecust.cn/pharmmapper/) [39].

### Networks Construction and Analysis

Before network construction, genes common to TNBC and tinengotinib were identified by Venny 2.1 (https://bioinfogp.cnb.csic.es/tools/venny/) [40]. The Venny is used to calculate intersection elements between two given lists, herein, the TNCB-genes and tinengotinib-target lists.

Cytoscape 3.2 software was used to construct and analyze three networks: the TNBC-gene network, the tinengotinib-target network, and the integrated TNBC–tinengotinib target network [41].

### Protein-Protein Interaction (PPI) Network

STRING database (https://string-db.org/) [42] was used to construct the PPI network of overlapping genes between TNBC and tinengotinib. We initially used the 62 common genes as inputs under the Multiple proteins by name search available in STRING. Under the same STRING tab, the organism was set to *Homo sapiens*, and the following advance settings were specified: full STRING network, a required score of 0.90 corresponding to interactions with the highest confidence and scientific evidence available and finally, FDR stringency of high (1%) were chosen. The generated network was exported to Cytoscape software for further analysis.

### Hub Genes Prediction, Gene Ontology (GO) and Pathway Enrichment Analysis

Prediction of core or hub genes from the PPI network was made using network validation and topological analysis offered by the cytoHubba plug-in in Cytoscape [43, 44]. Nodes were ranked by degree and the top 10 targets were designated as core targets. Subsequently, gene ontology analysis was made by ShinyGO 0.77 database (http://bioinformatics.sdstate.edu/go77/) [45]. Functional analysis encompasses three categories: biological processes (BPs), cellular components (CCs) and molecular functions (MFs). Furthermore, pathway enrichment analysis was conducted using the KEGG (Kyoto Encyclopedia of Genes and Genomes) resource integrated into ShinyGO. We considered a P-value cutoff (FDR) of 0.05 and the top 10 enriched functions were selected for analysis in each GO category.

### Molecular Docking

The top 10 core genes were first converted to corresponding Uniport proteins and then corresponding crystal structures of protein targets were retrieved from the RCSB database. Tinengotinib structure was downloaded from the PubChem database (CID: 137279257). UCSF Chimera was used to visualize and prepare the ligand and proteins before docking [46–48]. PDBs with reference ligands were used to define the binding site under each target correspondingly.

Docking was performed using the Autodock Vina extension on Chimera [49–52]. AMBER ff14SB force-field together with AM1-BCC model was deployed as charges model of standard residues and ligands respectively. Default parameters were used to rank docked conformations and an interaction zone of 5 Å was chosen for interaction analysis. Docked complexes were visualized by Discovery Studio Visualizer [53].

### Molecular Dynamics (MD) Simulation

MD simulations were conducted using the Particle Mesh Ewald MD (PMEMD) Compute Unified Device Architecture (PMEMD. CUDA) dual graphic processor unit (GPU) featured in the AMBER 18 package [54]. Utilizing the FF14SB AMBER force field for parameterization, the proteins were solvated and neutralized by adding hydrogen atoms, sodium ions, and counteracting chloride ions through the use of the LEAP module. Atomic solvation was achieved using a TIP3P box with 12 Å water molecules for each system. Partial minimization (2500 steps with 500 kcal/mol restraint potential) and full minimization (200 steps without conjugate restraint potential) were conducted [55]. Each system was then heated in a canonical ensemble (NVT) for 50 ps from 0 to 300 K using a Langevin thermostat that had a 1 ps random collision and a 10 kcal/mol Å harmonic potential restriction. Then, utilizing the SHAKE algorithm for the hydrogen bond constraint and the Barendsen-Barostat’s 1 bar pressure supply, equilibration was carried out. MD simulations were run in an isothermal-isobaric (NPT) ensemble at 300 K and a constant pressure of 1 bar for 200 ns with a 2-fs time interval [47, 55, 56]. Trajectories were saved every 1 ps and analysed using the CPTRAJ and PTRAJ modules of AMBER 18 GPU [57]. Discovery Studio Visualizer [53] and UCSF Chimera packages were used for visualization, structural and interaction analyses. Plots and statistical values were produced with the Origin program [55].

### Binding Free Energy (BFE) Calculations

Using the generalized Born surface area/molecular mechanics (MM/GBSA) technique, the free energy of binding (BFE) between tinengotinib against docked targets [58]. The MM/GBSA model predicts and evaluates the binding energies of molecular interactions in biological systems. MM/GBSA combines molecular mechanics computations with classical force fields to characterize molecular interactions, the generalized Born (GB) dielectric continuum solvent model, and surface area (SA) parameters to estimate the BFE. Internal energy, van der Waals interactions, electrostatic interactions, and other molecular forces were all included in the calculations of molecular mechanics.

The GB model took into account the atoms’ pairwise interactions and Born radii to predict the polar solvation-free energy. The surface tension constant (γ) was set at 0.0072 kcal/mol Å^2^, and the water probe radius of 1.4 Å was used to correlate the nonpolar solvation energy. This allowed the SA method to quantify the changes in hydrophobic interactions upon binding by calculating the buried surface area (BSA) during complex formation. The estimation of BFE was done with 50,000 MD trajectory frames [51, 55, 56]. The formula for BFE (∆G) is as follows:

where ΔG_bind_ signifies the gas-phase summation, E_gas_ is the gas-phase energy, G_sol_ is the free solvation energy, TΔS is the total interaction entropy, E_int_ is the internal energy, E_ele_ is the Coulomb energy, and E_vdw_ is the van der Waals energy. E_gas_ was calculated from the AMBER FF14SB force field, and G_sol_ was calculated from the energy contributions of polar and non-polar states.

## Results and Discussion

### Integrative Network Analysis of TNBC-Associated Genes and Tinengotinib Targets: A Systems Biology Approach

**Fig. 2:**
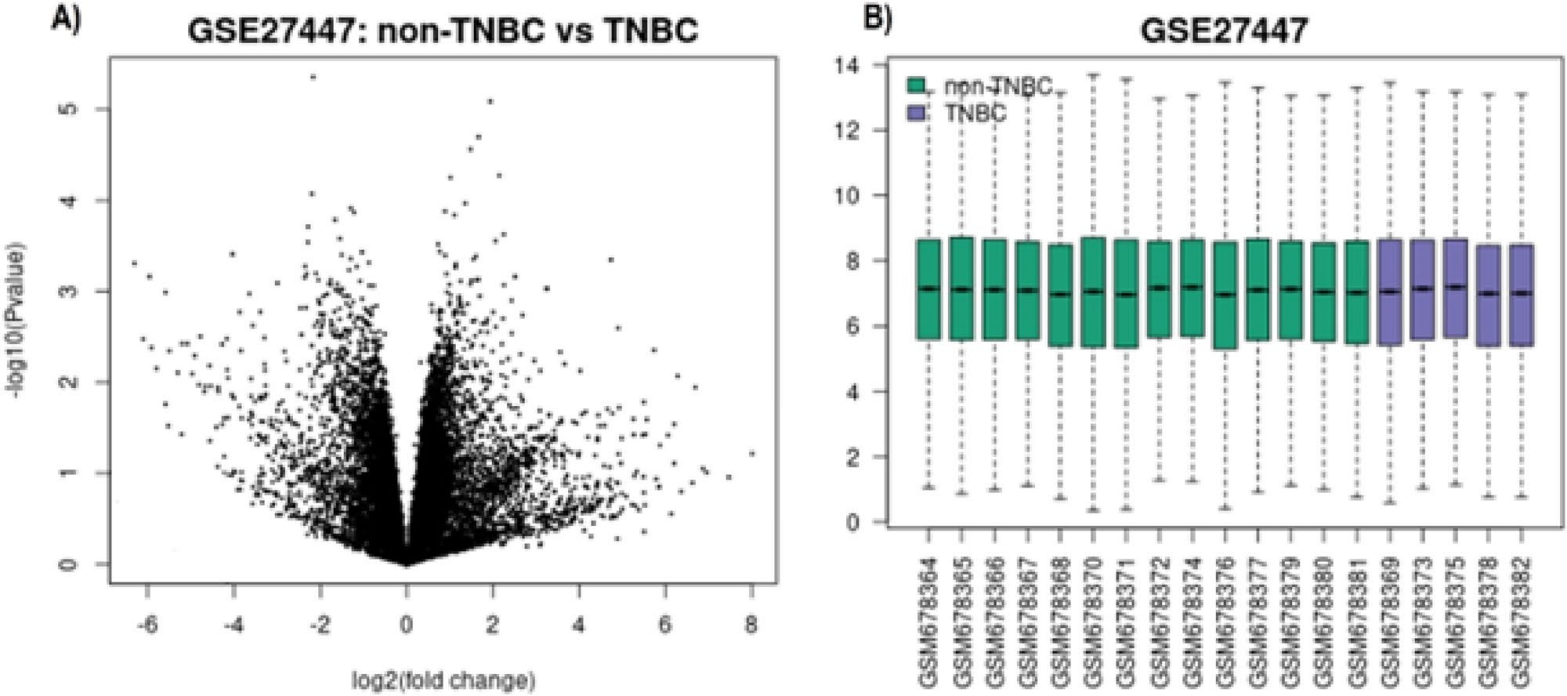
GSE27447 Summary Statistics: (A) Volcano Plot of Significant Genes, (B) Gene Expression Levels in TNBC vs. Non-TNBC Samples within GSE27447

The GEO dataset GSE27447 was analysed to explore the basic structure and distribution of gene expression data related to TNBC. Summary statistics (Fig. 2) showed that the adjusted p-values and log2 fold changes (log2FC) were distributed over a wide range, with some potential outliers observed.

Each data point in volcano plot (Fig. 2A) represents a gene, with its position determined by both the log2 fold change on the x-axis and the statistical significance p-value on the y-axis. Genes that appear far to the left or right (i.e., large fold change) and above the significance threshold are considered significantly downregulated or upregulated in TNBC. The boxplot (Fig. 2B) compares overall expression distributions across individual samples in both groups (non-TNBC in green and TNBC in purple).

A total of 85 genes were identified as significantly differentially expressed between control (non-TNBC) and test (TNBC) after applying the filtering criteria (adjusted p-value < 0.05 and |log2FC| >2) for the corresponding “upregulated” (positive log2 fold change) and “downregulated” (negative log2 fold change) genes.

TNBC-associated genes were further mapped from other databases, including, DISGENET, MalaCards, GeneCards, and Comparative Toxicogenomics Database. Under each database, ‘triple negative breast cancer’ was explicitly used for searching and protein-coding genes were only considered, yielding 10, 15, 2890, and 15 genes respectively, making a pool of 3015 genes (including the 85 deferentially expressed genes of the GSE dataset). Removing duplicates from the combined pool returned a unique list of 2930 TNBC-associated genes (supplementary file S1). On the other hand, a list of unique 144 targets (supplementary file S2) were predicted for tinengotinib using PubChem (8 targets), DGIdb (6 targets), GtoPdb (8 targets), SwissTargetPrediction (100 targets), and PharmMapper (22 targets), respectively.

**Fig. 3:**
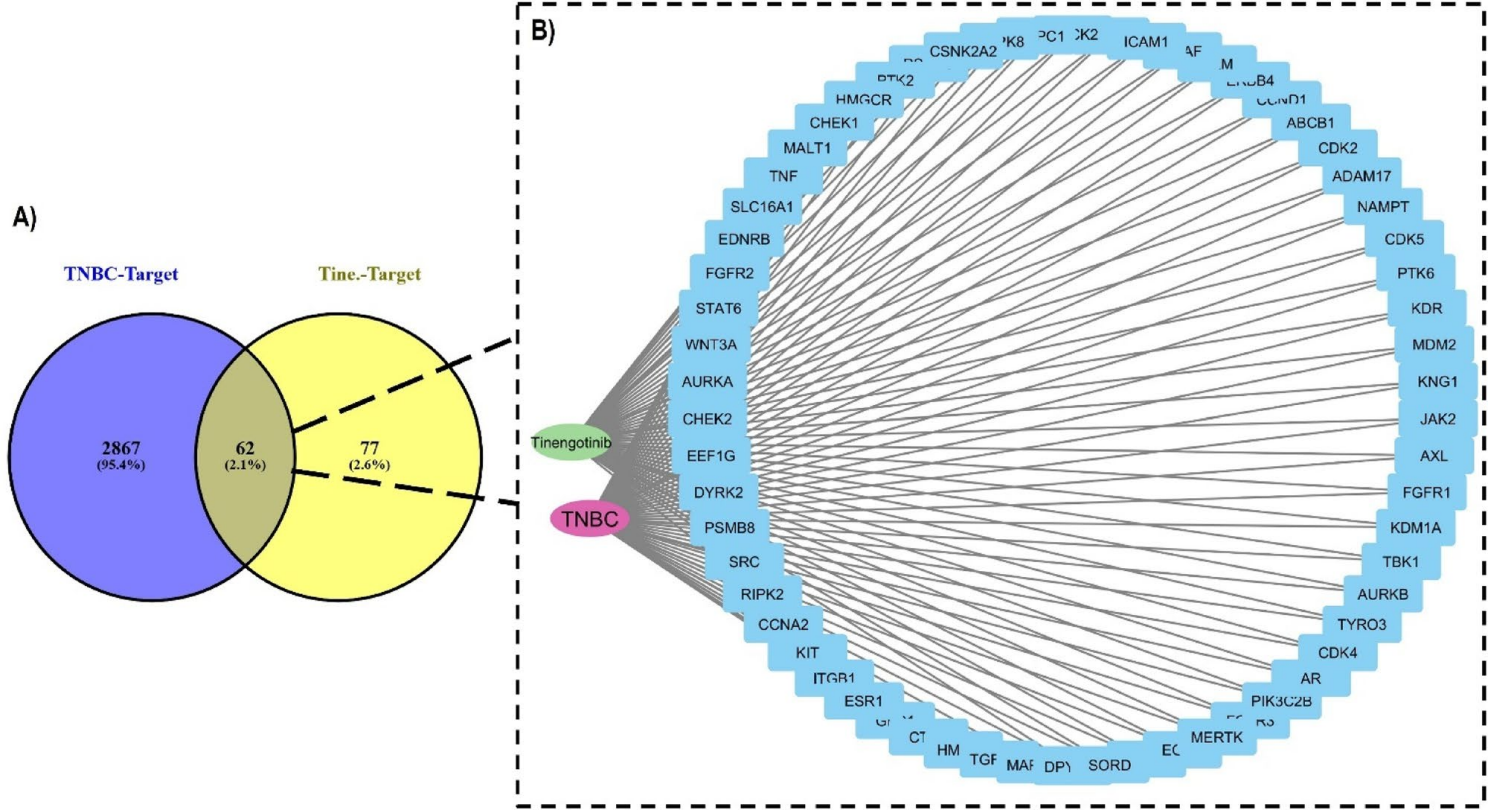
Genetic mapping and networks of TNBC and tinengotinib. (A) Common targets, (B) TNBC-tinengotinib combined network

Overlapping genes between TNBC and tinengotinib were identified by Venny 2.1 which accounts for the intersection elements across the two lists, arriving at 62 genes subsequently used to construct the TNBC-target-tinengotinib network (Fig. 3B) and subsequent PPI network (supplementary file S3). According to STRING, these genes are often aberrantly expressed or mutated, contributing to the aggressive clinical behavior observed in this subtype [59, 60]. Ranked by degree, proteins such as SRC (degree = 14), ESR1 [9], JAK2 [8], and EGFR [8] serve as major hubs, indicating their central roles in mediating extensive interactions within the network.

### Mapping Key Molecular Interactions and Functional Insights: PPI Network Construction, Hub Genes Prediction, and Pathway Enrichment Analysis

**Fig. 4:**
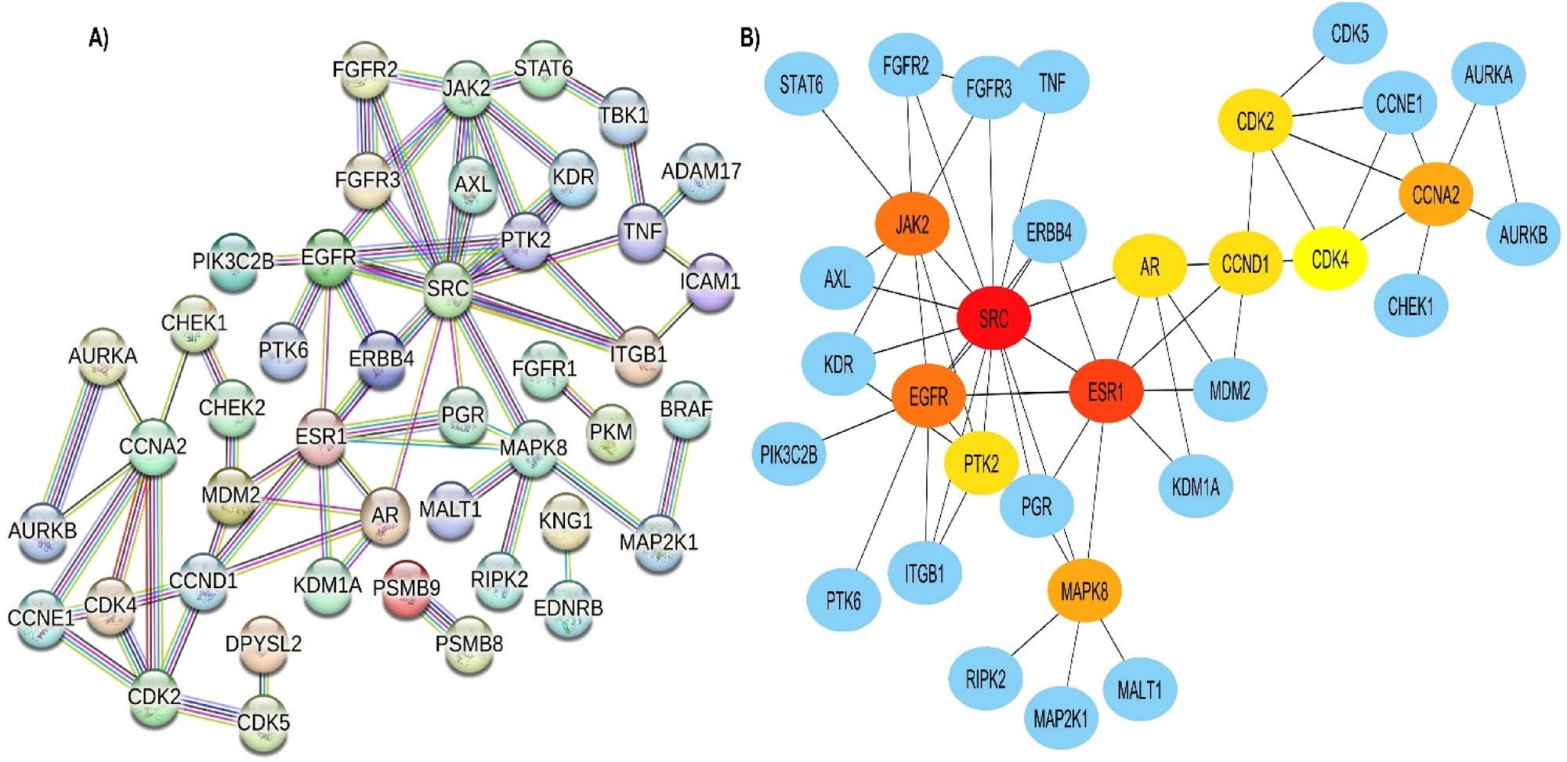
PPI network & top 10 genes. (A) Protein-protein interaction (PPI) network of common targets. Proteins are represented as nodes and their associations as edges. Colored nodes indicate query proteins and primary interactors. (B) cytoHubba ranking of PPI network with the color-scale for predicted hub genes, the darker the color, the higher the score

In this study, we used the STING tool to acquire the PPI network for the 62 overlapping targets. The network consisted of 62 nodes and 70 edges, with average node degree and neighbours of 2.26 and 3.526, respectively (Fig. 4A). In the network, nodes represent proteins, while edge thickness indicates the strength of the supporting data for the interaction.

The cytoHubba plug-in in Cytoscape software was used for validation and topological analysis of the PPI network; wherein, the top 10 nodes ranked by degree were predicted as core/hub targets (Fig. 4B). As can be depicted in Table 1, these hub genes play pivotal roles in various cellular processes, including signal transduction, cell cycle regulation, and apoptosis, and are often implicated in cancer development and progression.

The network appears as a predominantly unified cluster rather than fragmented modules, indicating that these genes participate in interrelated signalling pathways. The multi-colored edges underscore strong support from multiple data sources. From topological analyses, SRC, ESR1, JAK2, EGFR, and CCNA2, emerged as hubs with high-ranking scores. These proteins act as major regulatory nodes in cell signalling, cell cycle progression, and transcriptional control processes critically dysregulated in TNBC [61, 62]. The presence of cell-cycle–related genes such as CCND1, CCNA2, CDK2, CDK4, and MAPK8 (JNK1) in the core indicates that dysregulation of G1/S transition is central to TNBC progression and could be a targetable vulnerability [63]. Their interconnections with receptor tyrosine kinases (RTKs)-EGFR, FGFR1, FGFR2, FGFR3, ERBB4-emphasize a coordinated mechanism driving uncontrolled growth in TNBC.

**Table 1:** Predicted hub targets

No.		Gene	Score	UniProt ID	Protein Name	Description/Function
1	SRC		14	P12931	Proto-oncogene tyrosine-protein kinase	Non-receptor tyrosine kinase involved in the regulation of various cellular processes, including proliferation, differentiation, and survival.
2	ESR1		9	P03372	Estrogen receptor	Nuclear hormone receptor that binds estrogens and regulates gene expression involved in reproductive development and other cellular processes.
3	JAK2		8	O60674	Tyrosine-protein kinase JAK2	Non-receptor tyrosine kinase that transduces cytokine-mediated signals via the JAK-STAT pathway, influencing cell growth and hematopoiesis.
4	EGFR		8	P00533	Epidermal growth factor receptor	Receptor tyrosine kinase that binds EGF and regulates cell proliferation, differentiation, and survival; often implicated in cancer.
5	CCNA2		6	P20248	Cyclin-A2	Regulatory protein that controls cell cycle progression by activating cyclin-dependent kinases (CDKs) during S phase and G2/M transition.
6	MAPK8		6	P45983	Mitogen-activated protein kinase 8	Also known as JNK1, it is involved in stress-activated signaling pathways, influencing apoptosis and cellular responses to stress.
7	PTK2		5	Q05397	Focal adhesion kinase 1	Non-receptor protein tyrosine kinase involved in signaling pathways regulating cell adhesion, migration, and survival.
8	CCND1		5	P24385	G1/S-specific cyclin-D1	Regulatory component of CDK4/6, essential for cell cycle G1/S transition; overexpression linked to various cancers.
9	AR		5	P10275	Androgen receptor	Nuclear hormone receptor for androgens, regulating genes involved in male sexual development and reproductive function.
10	CDK2		5	P24941	Cyclin-dependent kinase 2	Serine/threonine kinase that, in association with cyclins, regulates cell cycle progression, particularly the G1 to S phase transition.

*Source: UniProt [64].

Tinengotinib, known for multi-kinase inhibition, may exert therapeutic benefits by targeting multiple RTKs simultaneously, potentially mitigating the resistance often observed with single-kinase inhibitors.

**Fig. 5:**
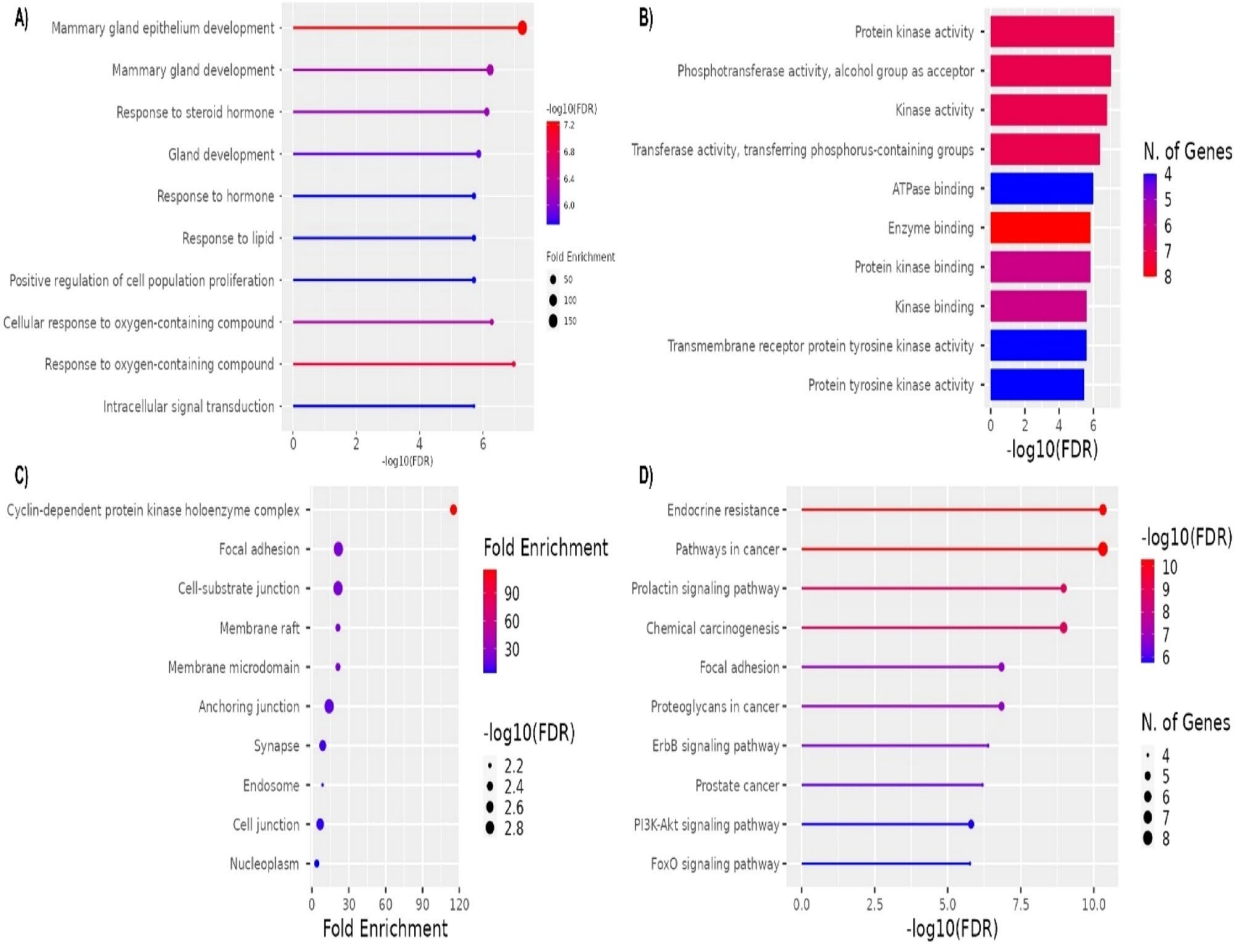
GO enrichment analysis: BP (A), MF (B), CC (C) and KEEG pathway (D)

The distribution of targets across growth factor receptors, cell cycle regulators, transcription factors, and stress-response kinases underscores the heterogeneous and multifaceted nature of TNBC. It supports the rationale for multi-kinase inhibition (as with tinengotinib) to disrupt several oncogenic pathways simultaneously.To gain more insights into the hub gene’s molecular functions and their involvement in complex TNBC pathogenesis, GO functional enrichment and KEGG pathway analysis were conducted in the ShinyGo database. GO enrichment analysis are summarized in Fig. 5.

**Fig. 6:**
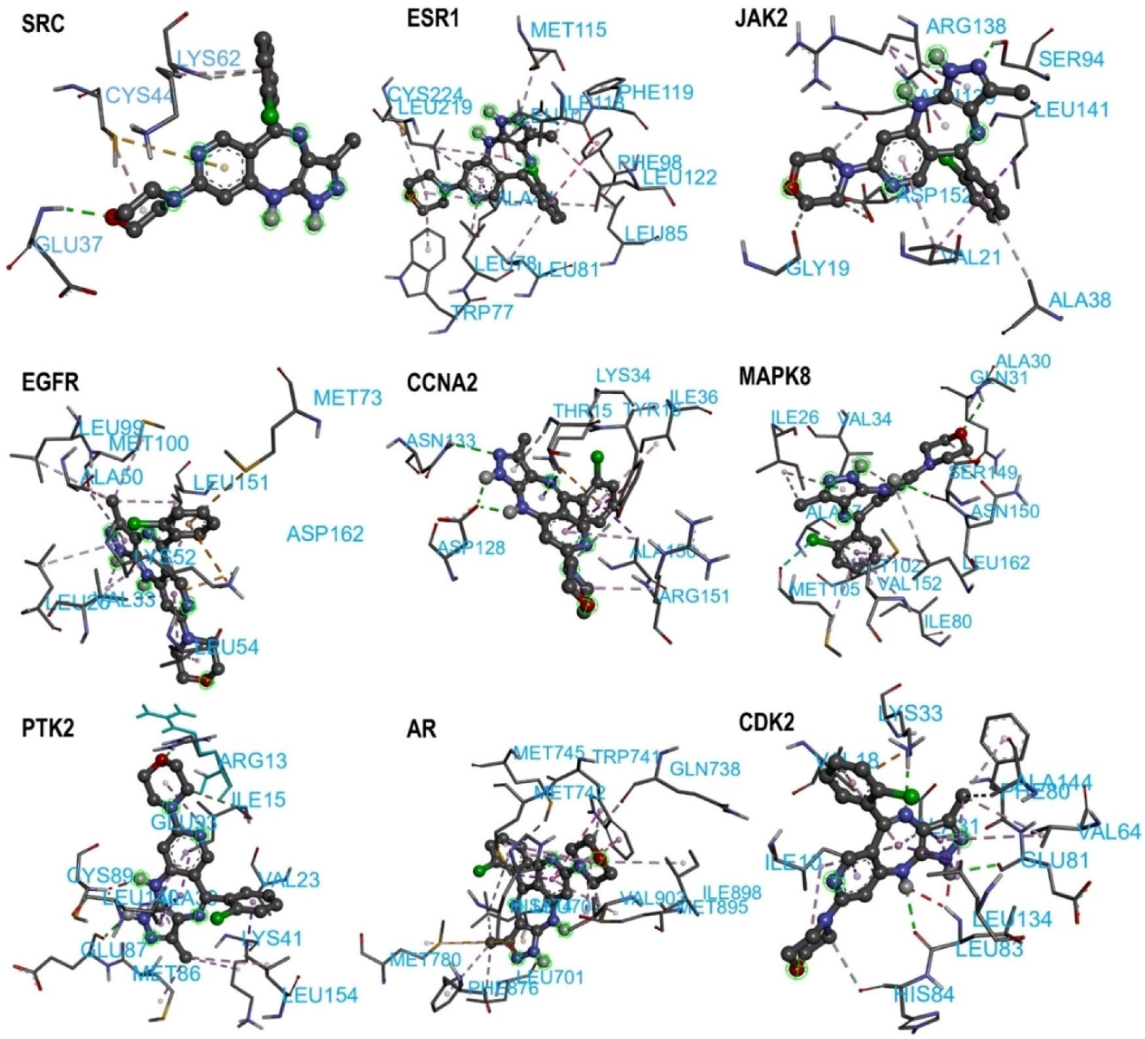
The 3D binding of tinengotinib across docked targets. Tinengotinib is given in ball and stick representation, binding residues in blue labelled sticks. Hydrophobic interactions are not shown

BP analysis highlighted key processes such as mammary gland epithelium development, hormone and lipid responses, and intracellular signal transduction, emphasizing the role of steroid hormone signalling and lipid metabolism in TNBC progression. These findings align with TNBC’s reliance on alternative signalling pathways despite its hormone receptor-negative status, as well as its metabolic adaptation to lipid-driven pathways for survival and proliferation [65, 66]. The CC analysis revealed significant enrichment in structures like the cyclin-dependent protein kinase (CDK) holoenzyme complex, focal adhesions, and membrane rafts. These components underscore the importance of cell cycle regulation, adhesion-mediated metastasis, and lipid-driven signalling in TNBC Fig. 6.

The CDK complex enrichment suggests potential therapeutic targeting of cell cycle dysregulation, while focal adhesion and membrane raft associations highlight TNBC’s aggressive metastatic behavior and reliance on membrane dynamics for survival [67, 68].

In MF analysis, kinase-related activities dominate, including protein kinase activity, phosphotransferase activity, and transmembrane receptor protein tyrosine kinase activity. These findings align with tinengotinib’s role as a multi-kinase inhibitor, targeting key signalling pathways such as EGFR, FGFR, and JAK2/STAT, which are critical for TNBC proliferation and resistance mechanisms [59, 65, 66].

The KEGG pathway analysis further supported these insights, identifying pathways like PI3K-Akt signalling, ErbB signalling, and focal adhesion as central to TNBC pathogenesis. The PI3K-Akt pathway is highly enriched, reflecting its role in TNBC cell survival and therapy resistance. Additionally, pathways such as endocrine resistance and prolactin signalling suggest compensatory mechanisms in TNBC that mimic hormone-driven growth, despite its hormone receptor-negative classification [69].

Collectively, these analyses highlighted tinengotinib’s potential to target TNBC’s kinase-driven signalling architecture, metabolic adaptations, and metastatic behavior.

### Tinengotinib Exhibited High Affinity and Stable Binding to Core Targets: Insights from Molecular Docking and Dynamics Simulations

Molecular docking is a commonly used technique for predicting and comparing the biological activity of molecules on various biological targets [70]. To further explore the interaction potential of tinengotinib with key TNBC-related targets, molecular docking was conducted using crystal structures obtained from the RCSB Protein Data Bank. Co-crystallized ligands in each structure were used as references to assess the reliability and relevance of docking results. The docking affinity scores, generated using AutoDock, represent the predicted binding strength between tinengotinib and each target protein, with more negative values indicating stronger binding. The corresponding binding affinities and 3D binding poses are provided in Table 2 and Fig. 6, respectively.

Among all targets, PTK2 (PDB ID: 6YOJ) showed the strongest binding affinity with tinengotinib at − 10.7 kcal/mol, while the reference ligand P4N exhibited an even stronger binding at − 12.6 kcal/mol, validating the structural suitability of the binding site. Similarly, MAPK8 (PDB ID: 4AWI) demonstrated a docking score of − 10.6 kcal/mol, close to its reference ligand AQ2 (–10.9 kcal/mol), suggesting comparable interaction potential. These results suggest that tinengotinib may effectively inhibit focal adhesion kinase (FAK/PTK2) and MAPK8, both of which play significant roles in cancer cell signaling and microtubule dynamics in TNBC [71].

Other key proteins also showed strong binding affinities. EGFR (PDB ID: 4WKQ) and ESR1 (PDB ID: 1XP1) yielded docking scores of − 9.5 kcal/mol and − 9.4 kcal/mol, respectively, while their reference ligands IRE and AIH demonstrated binding scores of − 9.5 kcal/mol and − 12.3 kcal/mol, respectively. This implies that tinengotinib may disrupt EGFR signaling, a central pathway in TNBC progression, and possibly modulate ER-associated pathways, despite TNBC typically lacking ER expression [72].

**Table 2:** Tinengotinib Docking against hub genes

Gene	PDBID	ligand	Affinity (Kcal/mol)
SRC	1O4A	Tinengotinib Compound 197	-6.3 -7.2
ESR1	1XP1	Tinengotinib AIH	-9.4 -12.3
JAK2	6BBV	Tinengotinib D7D	-8.8 -8.8
EGFR	4WKQ	Tinengotinib IRE	-9.5 -9.5
CCNA2	7RWF	Tinengotinib 7TW	-6.0 -11.6
MAPK8	4AWI	Tinengotinib AQ2	-10.6 -10.9
PTK2	6YOJ	Tinengotinib P4N	-10.7 -12.6
CCND1	9CSK	Tinengotinib A1AZ4	-9.3 -9.7
AR	3B66	Tinengotinib B66	− 4.1 − 4.7
CDK2	4FKL	Tinengotinib CK2	-8.5 -7.2

JAK2 (PDB ID: 6BBV) and CDK2 (PDB ID: 4FKL) showed moderate docking affinities of − 8.8 kcal/mol and − 8.5 kcal/mol, respectively. Their co-crystallized ligands (D7D and CK2) returned similar or slightly stronger scores, highlighting the potential of tinengotinib to interfere with cytokine signaling and cell cycle regulation via these kinases [73]. In contrast, AR (PDB ID: 3B66) exhibited the weakest interaction with tinengotinib (–4.1 kcal/mol), significantly lower than its reference ligand (–4.7 kcal/mol), indicating minimal potential for androgen receptor inhibition. This aligns with the limited relevance of AR as a therapeutic target for tinengotinib in TNBC.

Visualized through 2D interaction diagrams (Table 3), the analysis confirms that hydrophobic interactions such as van der Waals forces, alkyl, and pi-alkyl interactions dominate across most targets. Residues like LEU, PHE, ILE, MET, and VAL are recurrently involved, suggesting that tinengotinib consistently engages conserved hydrophobic pockets within the target proteins—typical of many oncogenic kinases and receptors. In addition to hydrophobic forces, hydrogen bonds play a crucial role in stabilizing ligand-protein complexes. These were especially evident in targets such as CCNA2, JAK2, PTK2, SRC, and EGFR, reinforcing the specificity and directionality of binding. For example, in JAK2 and SRC complexes, residues such as GLY, ASN, and SER contributed hydrogen bonds that anchor the ligand within the active site.

**Table 3:**
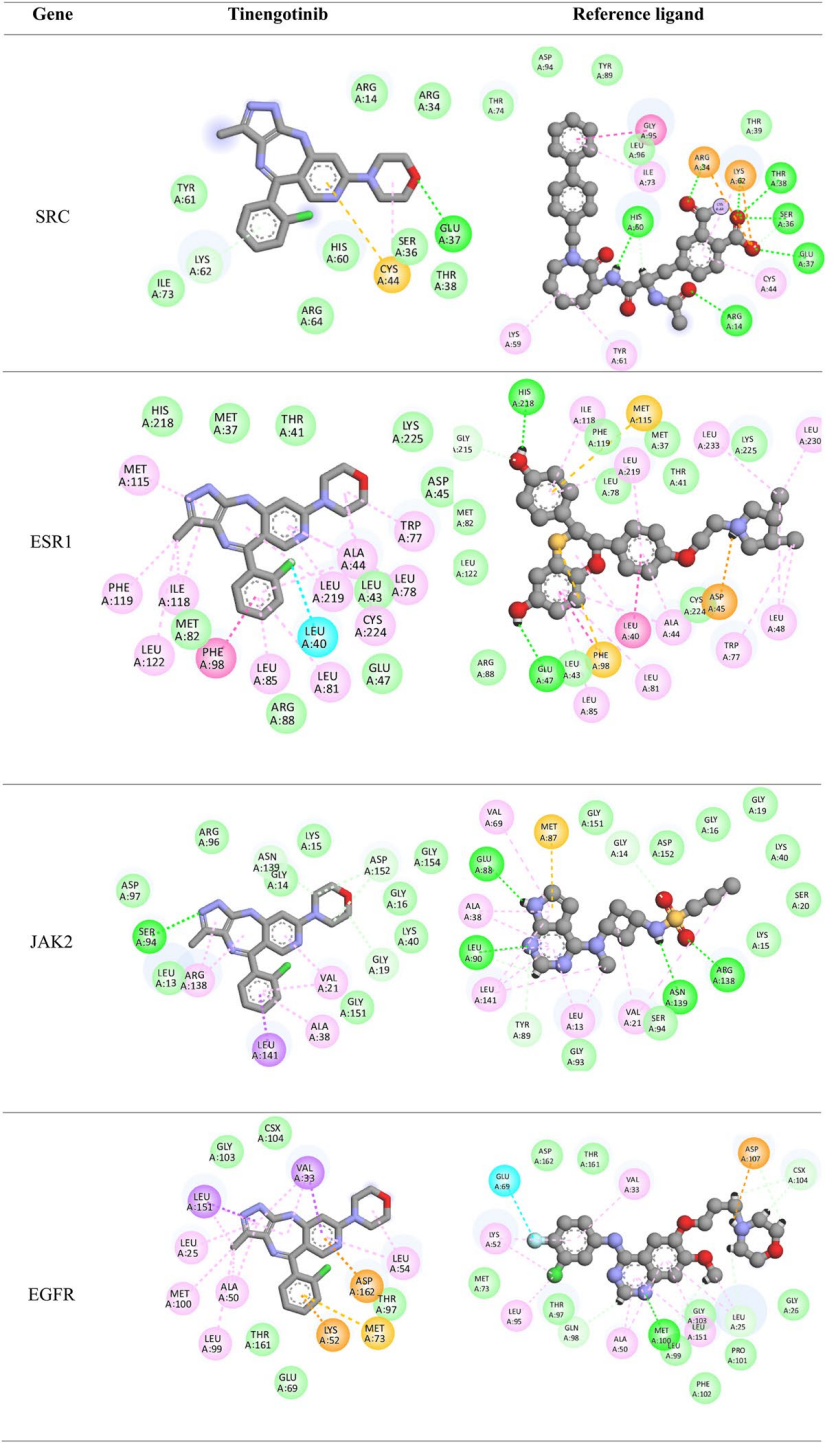

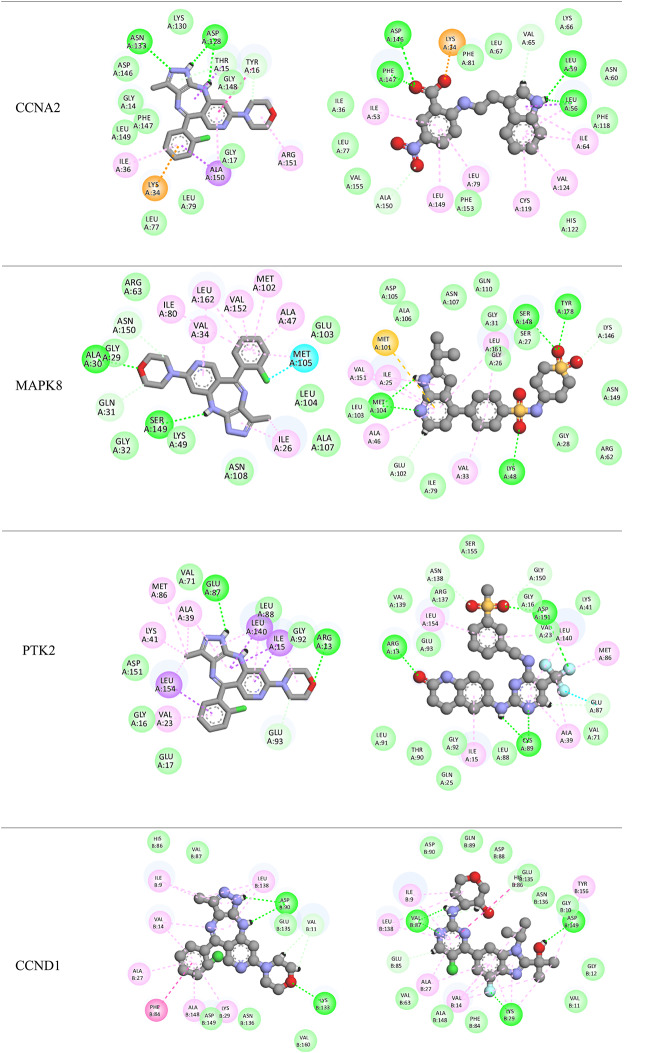

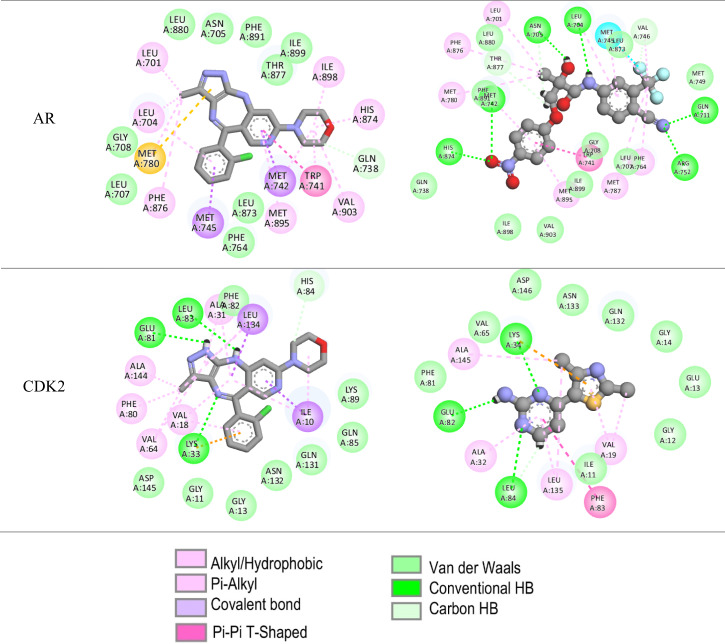
2D Docking poses

Pi-based interactions provide further stabilization and possibly contribute to target selectivity. These include pi–sulfur interactions in EGFR, pi–pi T-shaped stacking in ESR1, and pi–alkyl interactions in multiple kinases including CCND1 and MAPK8. Furthermore, halogen bonding, typically a rarer and more directional interaction, was observed in complexes with MAPK8 and ESR1, involving residues such as GLU and LEU. These interactions are likely facilitated by the halogen moieties of tinengotinib and may contribute to both binding affinity and selectivity.

Interestingly, comparison with co-crystallized reference ligands revealed that tinengotinib engages several of the same residues, suggesting a degree of mimicry in binding orientation, particularly within ATP-binding sites of kinases. In EGFR (PDB: 4WKQ), tinengotinib and the reference ligand (IRE) both occupy the ATP-binding cleft, making key contacts with MET, ASP, and PHE. In PTK2 (PDB: 6YOJ), tinengotinib forms similar interactions as the reference ligand P4N, including hydrogen bonds with GLY and ARG, and hydrophobic interactions with LEU and ILE residues. ESR1 (PDB: 1XP1) displayed strong affinity and selectivity, supported by π-interactions and halogen bonding, which were consistent with those formed by the native ligand AIH.

The docking affinities (Table 2) further support the binding interaction findings, with PTK2 (-10.7 kcal/mol) and MAPK8 (-10.6 kcal/mol) demonstrating the strongest binding to tinengotinib. These scores closely approach those of their co-crystallized ligands (-12.6 and − 10.9 kcal/mol respectively), underscoring tinengotinib’s potential as a competitive inhibitor. EGFR and ESR1 also showed high binding affinities (-9.5 and − 9.4 kcal/mol), pointing toward the compound’s ability to disrupt receptor tyrosine kinase and hormone receptor pathways in TNBC. In contrast, weaker interactions were observed with AR (-4.1 kcal/mol), indicating that androgen receptor signaling may not be a primary target. This broad interaction spectrum suggests a capacity to simultaneously disrupt oncogenic kinase signaling and hormonal pathways, offering therapeutic promise against triple-negative breast cancer (TNBC).

Five proteins-PTK2, MAPK8, EGFR, ESR1, and SRC-were selected for MD simulations based on a combined assessment of binding affinity and network importance. PTK2 and MAPK8 showed the strongest docking scores (–10.7 and − 10.6 kcal/mol), indicating strong binding with tinengotinib. EGFR and ESR1 were chosen for their high binding affinities and central roles in TNBC pathways. SRC, despite a lower docking score (–6.3 kcal/mol), ranked highest in network centrality, reflecting its functional importance.

**Fig. 7:**
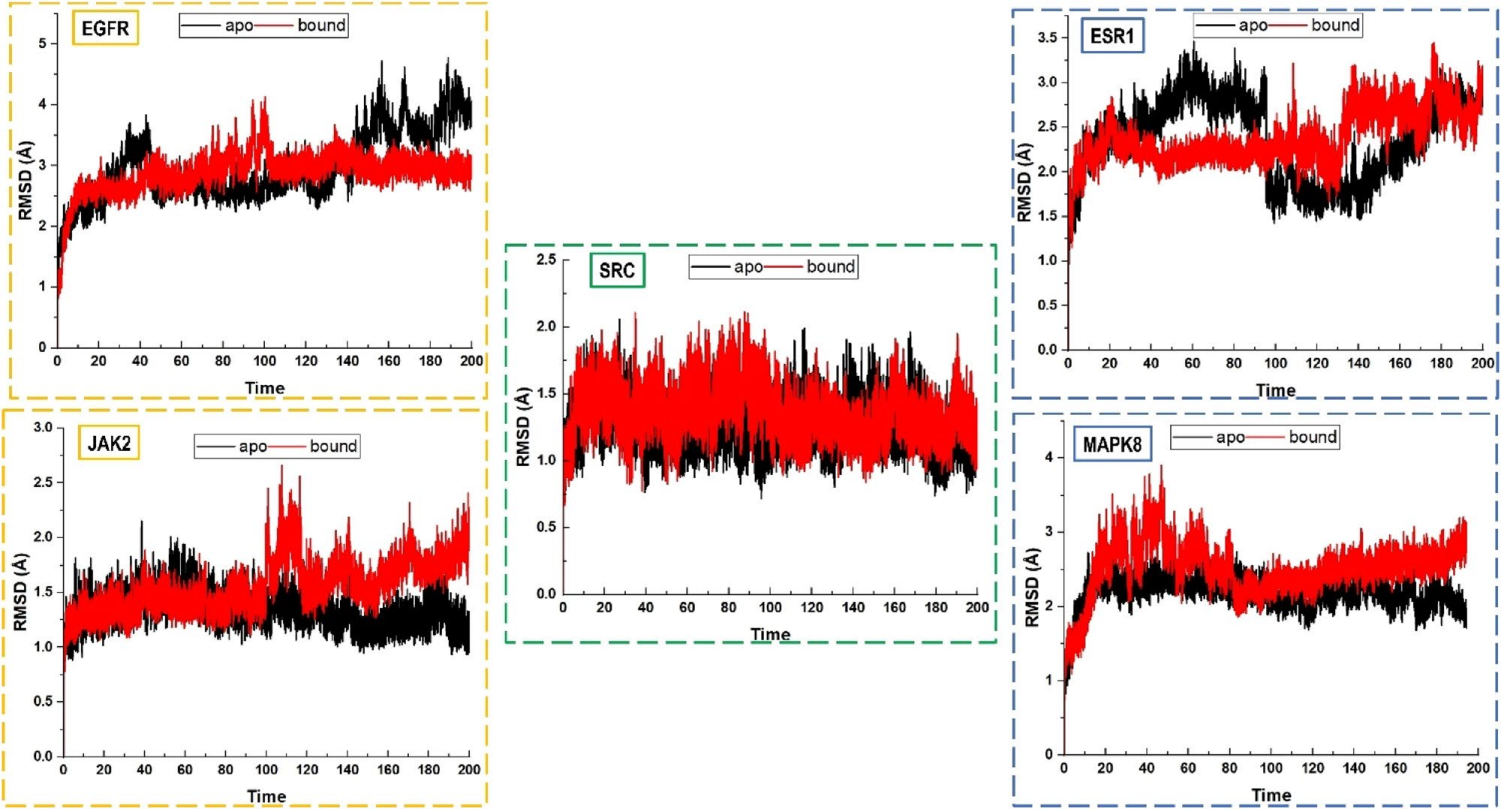
RMSD plots of five top-ranked hub proteins. Time is measured in nanoseconds

To evaluate the stability of tinengotinib–protein complexes, MD simulations were performed on five prioritized targets (EGFR, ESR1, PTK2, MAPK8, and SRC). Root Mean Square Deviation (RMSD) was analyzed to monitor structural fluctuations of apo and bound systems over 200 ns (Fig. 7) [74].

Overall, the RMSD trajectories remained within a stable range (~ 1.5–3.5 Å), indicating that tinengotinib binding did not disrupt the global protein folds [74]. EGFR and ESR1 exhibited slightly elevated RMSD in the bound state compared to apo, suggesting local rearrangements or induced-fit adaptations within the binding site, a common feature in kinase–inhibitor interactions [75]. PTK2 displayed the most notable stabilization upon binding, with the ligand-bound form showing lower RMSD fluctuations than apo, consistent with tight accommodation of tinengotinib. MAPK8 showed higher initial deviations in the bound state but stabilized after ~ 60 ns, reflecting a dynamic yet stable binding mode. In contrast, SRC maintained nearly identical apo and bound RMSD trajectories, further supporting stable ligand accommodation without major conformational shifts.

Collectively, these results demonstrate that tinengotinib binding confers structural stability across multiple TNBC-related kinases, supporting its potential as a multi-target inhibitor with stable protein–ligand interactions.

**Fig. 8:**
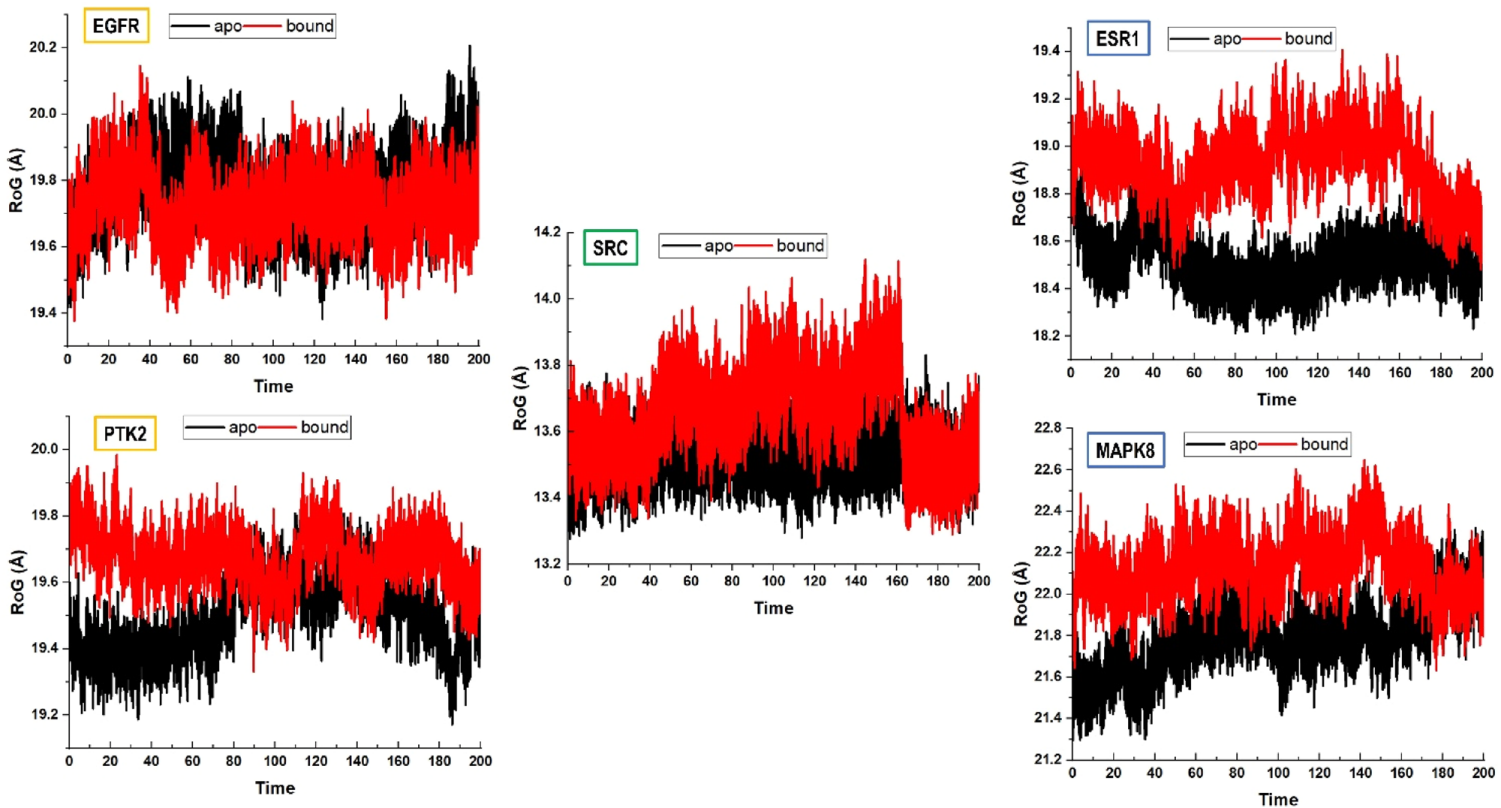
Radius of gyration (RoG) profiles of apo (black) and tinengotinib-bound (red) forms of EGFR, ESR1, PTK2, MAPK8, and SRC during 200 ns molecular dynamics simulations

The RoG profiles (Fig. 8) revealed that structural compactness was largely maintained across the simulations, with average values around 22–28 Å. However, subtle differences were noted between apo and tinengotinib-bound systems. As shown in Fig. 8, all five proteins displayed consistent RoG values throughout the 200 ns simulations, confirming that both apo and bound systems remained structurally stable without significant unfolding events.

**Fig. 9:**
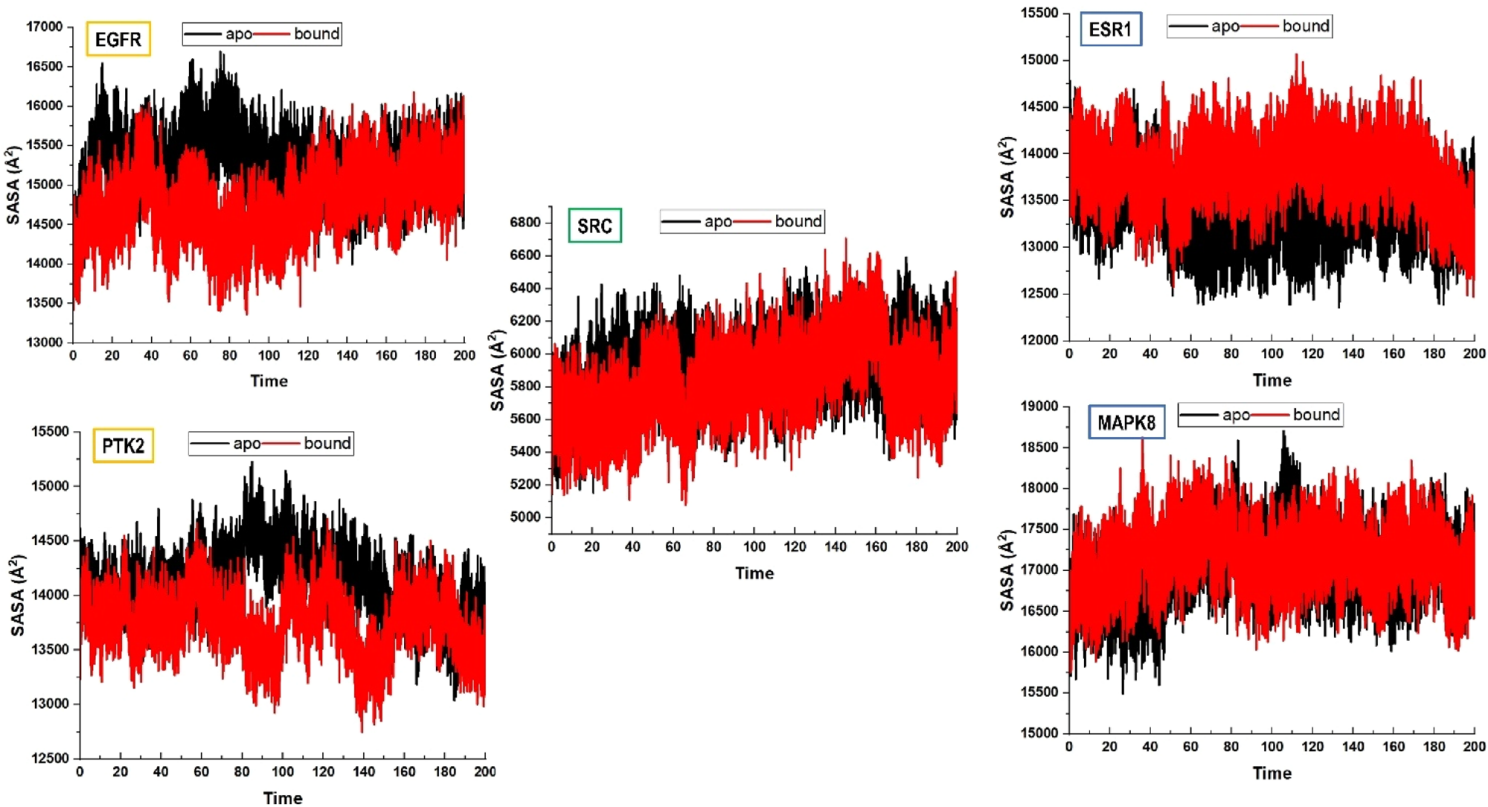
Solvent accessible surface area (SASA) analysis of apo and tinengotinib-bound protein systems. The plots depict target-specific variations in solvent exposure, where decreases in SASA (EGFR and PTK2) indicate structural compaction upon ligand binding, while increases (ESR1, SRC, and MAPK8) reflect conformational expansion.Time is measured in nanoseconds

Notably, the bound complexes of EGFR, ESR1, PTK2, MAPK8, and SRC exhibited slightly elevated RoG values compared to their apo counterparts, indicating minor conformational expansion upon ligand association. This observation suggests that tinengotinib binding introduces subtle structural rearrangements, while still maintaining the global integrity of the proteins. The results align with the RMSD analysis, further supporting the dynamic stability of the ligand–protein complexes. To further investigate the conformational effects of Tinengotinib binding, the solvent-accessible surface area (SASA) was evaluated for the apo and ligand-bound systems across the five protein targets. The SASA values are indicative of folding and unfolding events, reflecting compactness and conformational flexibility during the simulation. the results reveal distinct binding-induced effects on solvent exposure, As depicted in Fig. 9.

In the case of EGFR and PTK2, tinengotinib binding reduced SASA values compared to the apo systems, indicating structural compaction and stabilization through decreased solvent accessibility. By contrast, ESR1, SRC, and MAPK8 exhibited an increase in SASA upon ligand binding, suggesting expansion of solvent-exposed regions, potentially reflecting conformational rearrangements triggered by the interaction. Notably, the increase was most pronounced for ESR1, consistent with significant reorganization of its binding cavity. Taken together, these findings highlight that tinengotinib exerts target-specific effects on protein dynamics: promoting folding and compactness in some systems, while inducing expansion in others.

To evaluate the binding affinity of tinengotinib with the selected kinase targets, MM/GBSA binding free energy calculations were carried out over 50,000 frames of the stabilized trajectories (Table 4). Among the investigated systems, MAPK8 (-45.71 kcal/mol) and ESR1 (-44.21 kcal/mol) displayed the most favorable ΔG_total values, indicating stronger binding affinity compared to PTK2 (-39.11 kcal/mol), EGFR (-23.23 kcal/mol), and SRC (-21.48 kcal/mol).

**Table 4:** Binding free energy terms of Tinengotinib complexes with selected protein targets expressed in kcal/mol with the standard error of mean

Energy components (kcal/mol)							
System	**ΔE**_**vdW**_	**ΔE**_**ele**_	**ΔG**_**GB**_	**ΔG**_**SA**_	**ΔG**_**gas**_	**ΔG**_**sol**_	**ΔG**_**bind**_
SRC	-29.75 ± 0.11	-6.46 ± 0.16	18.20 ± 0.14	-3.46 ± 0.01	-36.21 ± 0.22	14.73 ± 0.13	-21.48 ± 0.12
PTK2	-47.14 ± 0.09	-15.09 ± 0.10	28.65 ± 0.09	-5.52 ± 0.01	-62.23 ± 0.14	23.13 ± 0.08	-39.11 ± 0.09
MAPK8	-48.07 ± 0.13	-17.59 ± 0.10	25.46 ± 0.07	-5.51 ± 0.01	-65.66 ± 0.14	19.95 ± 0.07	-45.71 ± 0.15
ESR1	-53.75 ± 0.09	-8.53 ± 0.10	24.49 ± 0.07	-6.41 ± 0.01	-62.29 ± 0.15	18.08 ± 0.07	-44.21 ± 0.12
EGFR	-35.39 ± 0.14	-13.55 ± 0.20	30.07 ± 0.20	-4.36 ± 0.02	-48.95 ± 0.27	25.71 ± 0.19	-23.23 ± 0.12

*∆E*_*elec*_*(electrostatic energy)*, * ∆E*_*vdW*_*(van der Waals energy)*, * ∆G*_*GB*_*(polar solvation energy)*, * ∆G*_*SA*_*(non-polar solvation energy)*, * ∆G*_*gas*_*(gas-phase energy)*, * ∆G*_*solv*_*(Total solvation free energy of polar and non-polar states)*, * and ∆G*_*bind*_*(total free binding energy)*

Analysis of the energy components showed that van der Waals interactions were the dominant stabilizing factor across all complexes. Electrostatic interactions further enhanced stability in MAPK8 and PTK2, while the polar solvation term consistently opposed binding. In contrast, the nonpolar solvation term contributed modestly but favorably to the overall interaction.

## Conclusion

Triple-negative breast cancer (TNBC) is an aggressive and molecularly diverse subtype lacking hormone receptors and HER2 amplification, which significantly limits treatment options and worsens clinical outcomes. Tinengotinib (TT-00420), a spectrum-selective multi-kinase inhibitor, shows promise for targeting multiple dysregulated pathways implicated in TNBC progression. In this study, we employed an integrative computational approach—comprising network pharmacology, molecular docking, and molecular dynamics simulations—to elucidate the potential mechanism of action of tinengotinib in TNBC. Molecular docking and MMGBSA analyses suggested that tinengotinib interacts with several key signaling pathways, including PI3K-Akt, ErbB, and focal adhesion, and binds stably to critical proteins such as PTK2, MAPK8, EGFR, and ESR1, which may underlie its anti-tumor potential. Molecular dynamics simulations further revealed that tinengotinib stabilizes these protein-ligand complexes, inducing protein-specific conformational changes, including compaction in EGFR and PTK2 and expansion in MAPK8 and ESR1, reflecting its versatile binding effects. These results collectively suggest that tinengotinib exerts its anticancer effects by simultaneously modulating multiple signaling nodes central to TNBC progression and drug resistance.

While the computational evidence is encouraging, experimental studies are essential to substantiate tinengotinib’s role as a multi-targeted therapeutic strategy for TNBC. Future research including biochemical assays, cellular studies, and animal models can validate the functional relevance of tinengotinib’s interactions and assess its therapeutic efficacy and safety in a biological context, supporting the immediate translational applicability of these findings. Moreover, exploring resistance mechanisms and combination therapies could further enhance its clinical potential.

## Supplementary Information

Below is the link to the electronic supplementary material.

Supplementary Material 1

Supplementary Material 2

Supplementary Material 3

Supplementary Material 4

## Data Availability

All data can be made available upon request to the Corresponding Author.
