# Supplementary material for: Unravelling Tinengotinib’s Mechanistic Landscape in Triple-Negative Breast Cancer Via Network Pharmacology and in Silico Simulation Techniques
Source: Cell Biochem Biophys. 2025 Oct 16;84(1):873–89. doi: 10.1007/s12013-025-01907-y (PMC12967403; doi:10.1007/s12013-025-01907-y)
Supplement: Supplementary file 3 — Supplementary Material 3 [file 12013_2025_1907_MOESM3_ESM.docx]

**Table S3:** STRING PPI network analysis.

| Gene | Degree | Eccentricity | Connectivity | Radiality | TopologicalCoefficient |
| --- | --- | --- | --- | --- | --- |
| SRC | 14 | 5 | 4.7 | 0.9 | 0.2 |
| ESR1 | 9 | 4 | 5.6 | 0.9 | 0.2 |
| JAK2 | 8 | 6 | 5 | 0.9 | 0.3 |
| EGFR | 8 | 5 | 5.6 | 0.9 | 0.3 |
| CCNA2 | 6 | 6 | 3 | 0.8 | 0.4 |
| MAPK8 | 6 | 5 | 5 | 0.9 | 0.3 |
| CCND1 | 5 | 4 | 5.4 | 0.9 | 0.4 |
| AR | 5 | 4 | 6.8 | 0.9 | 0.3 |
| CDK2 | 5 | 5 | 4 | 0.8 | 0.3 |
| PTK2 | 5 | 6 | 7.4 | 0.9 | 0.4 |
| MDM2 | 4 | 4 | 5.3 | 0.9 | 0.4 |
| CDK4 | 4 | 5 | 4.8 | 0.8 | 0.4 |
| TNF | 4 | 6 | 4.8 | 0.9 | 0.3 |
| ITGB1 | 4 | 6 | 7.3 | 0.9 | 0.4 |
| CCNE1 | 3 | 6 | 5 | 0.8 | 0.6 |
| KDR | 3 | 6 | 9 | 0.9 | 0.6 |
| ERBB4 | 3 | 5 | 10.3 | 0.9 | 0.5 |
| PGR | 3 | 5 | 9.7 | 0.9 | 0.5 |
| FGFR3 | 3 | 6 | 8.3 | 0.9 | 0.6 |
| FGFR2 | 3 | 6 | 8.3 | 0.9 | 0.6 |
| AURKA | 2 | 7 | 4 | 0.7 | 0.7 |
| AURKB | 2 | 7 | 4 | 0.7 | 0.7 |
| CHEK2 | 2 | 5 | 3 | 0.8 | 0.5 |
| ICAM1 | 2 | 7 | 4 | 0.8 | 0.6 |
| CDK5 | 2 | 6 | 3 | 0.8 | 0.5 |
| STAT6 | 2 | 7 | 5 | 0.8 | 0.5 |
| TBK1 | 2 | 7 | 3 | 0.8 | 0.5 |
| AXL | 2 | 6 | 11 | 0.8 | 0.7 |
| MAP2K1 | 2 | 6 | 3.5 | 0.8 | 0.5 |
| KDM1A | 2 | 5 | 7 | 0.8 | 0.8 |
| CHEK1 | 2 | 6 | 4 | 0.8 | 0.5 |
| RIPK2 | 1 | 6 | 6 | 0.8 | 0.0 |
| PIK3C2B | 1 | 6 | 8 | 0.8 | 0.0 |
| PTK6 | 1 | 6 | 8 | 0.8 | 0.0 |
| BRAF | 1 | 7 | 2 | 0.8 | 0.0 |
| ADAM17 | 1 | 7 | 4 | 0.8 | 0.0 |
| PKM | 1 | 1 | 1 | 1.0 | 0.0 |
| FGFR1 | 1 | 1 | 1 | 1.0 | 0.0 |
| PSMB9 | 1 | 1 | 1 | 1.0 | 0.0 |
| PSMB8 | 1 | 1 | 1 | 1.0 | 0.0 |
| EDNRB | 1 | 1 | 1 | 1.0 | 0.0 |
| KNG1 | 1 | 1 | 1 | 1.0 | 0.0 |
| MALT1 | 1 | 6 | 6 | 0.8 | 0.0 |
| DPYSL2 | 1 | 7 | 2 | 0.7 | 0.0 |
| GLO1 | 0 | 0 | 0 | Infinity | 0.0 |
| WNT3A | 0 | 0 | 0 | Infinity | 0.0 |
| ROCK2 | 0 | 0 | 0 | Infinity | 0.0 |
| NAMPT | 0 | 0 | 0 | Infinity | 0.0 |
| SLC16A1 | 0 | 0 | 0 | Infinity | 0.0 |
| TYRO3 | 0 | 0 | 0 | Infinity | 0.0 |
| CSNK2A2 | 0 | 0 | 0 | Infinity | 0.0 |
| EEF1G | 0 | 0 | 0 | Infinity | 0.0 |
| MERTK | 0 | 0 | 0 | Infinity | 0.0 |
| CTSB | 0 | 0 | 0 | Infinity | 0.0 |
| HMGCR | 0 | 0 | 0 | Infinity | 0.0 |
| PABPC1 | 0 | 0 | 0 | Infinity | 0.0 |
| DYRK2 | 0 | 0 | 0 | Infinity | 0.0 |
| ABCB1 | 0 | 0 | 0 | Infinity | 0.0 |
| HMOX1 | 0 | 0 | 0 | Infinity | 0.0 |
| KIT | 0 | 0 | 0 | Infinity | 0.0 |
| TGFBR1 | 0 | 0 | 0 | Infinity | 0.0 |
| SORD | 0 | 0 | 0 | Infinity | 0.0 |

The Cytoscape analysis of the STRING-derived PPI network for the 62 overlapping targets between TNBC and tinengotinib reveals a hierarchy of connectivity among nodes. Proteins such as SRC (degree = 14), ESR1 (9), JAK2 (8), and EGFR (8) serve as major hubs, indicating their central roles in mediating extensive interactions within the network.

`

Their high degree values and relatively low eccentricities (4–6) highlight their proximity to other proteins, suggesting they act as information relay centers in the network topology. Additionally, these hub proteins display moderate topological coefficients (0.2–0.3), implying a diverse neighborhood of interaction partners rather than participation in tightly-knit cliques. The radiality values, generally ranging between 0.8 and 0.9, indicate strong overall centrality across most targets. Notably, proteins such as PTK2, although having a moderate degree (5), display high connectivity (7.4) and radiality (0.9), positioning them as potentially influential nodes despite fewer direct connections.

Conversely, a significant subset of proteins (e.g., GLO1, ROCK2, TYRO3, CTSB) exhibit zero connectivity (degree = 0), eccentricity = 0, and infinite radiality, indicating that these nodes are completely disconnected from the main PPI network and do not participate in any observed interactions. This dichotomy between highly connected hubs and isolated nodes reflects the functional heterogeneity of the protein set, where certain genes may be core to TNBC signaling or drug response, while others may be less functionally integrated or context dependent. Overall, the topology supports the interpretation that the most connected proteins—particularly SRC, ESR1, EGFR, and JAK2—serve as key regulatory elements and likely represent critical points of tinengotinib’s therapeutic action in TNBC.
