# Supplementary material for: Unravelling Tinengotinib’s Mechanistic Landscape in Triple-Negative Breast Cancer Via Network Pharmacology and in Silico Simulation Techniques
Source: Cell Biochem Biophys. 2025 Oct 16;84(1):873–89. doi: 10.1007/s12013-025-01907-y (PMC12967403; doi:10.1007/s12013-025-01907-y)
Supplement: Supplementary file 4 — Supplementary Material 4 [file 12013_2025_1907_MOESM4_ESM.docx]

**Table S4:** GO and KEGG enrichment analysis of core targets.

| GO Category | Enrichment FDR | nGenes | Fold  Enrichment | Pathway | Genes |  |  |  |  |  |
| --- | --- | --- | --- | --- | --- | --- | --- | --- | --- | --- |
|  | 5.65E-08 | 5 | 173.4855 | Mammary gland epithelium development | ESR1 JAK2 CCND1 AR SRC | | | | | |
|  | 1.06E-07 | 9 | 12.44323 | Response to oxygen-containing compound | ESR1 JAK2 MAPK8 CCND1 CDK2 EGFR AR PTK2 SRC | | | | | |
|  | 5.23E-07 | 8 | 15.43268 | Cellular response to oxygen-containing compound | ESR1 JAK2 MAPK8 CDK2 EGFR AR PTK2 SRC | | | | | |
|  | 5.88E-07 | 5 | 83.31871 | Mammary gland development | ESR1 JAK2 CCND1 AR SRC | | | | | |
| BP | 7.53E-07 | 6 | 37.52428 | Response to steroid hormone | ESR1 JAK2 CCND1 EGFR AR SRC | | | | | |
|  | 1.36E-06 | 6 | 32.96602 | Gland development | ESR1 JAK2 CCND1 EGFR AR SRC | | | | | |
|  | 1.91E-06 | 7 | 17.01557 | Positive regulation of cell population proliferation | ESR1 JAK2 CCND1 CDK2 EGFR AR PTK2 | | | | | |
|  | 1.91E-06 | 7 | 17.76575 | Response to hormone | ESR1 JAK2 CCND1 EGFR AR PTK2 SRC | | | | | |
|  | 1.91E-06 | 7 | 17.43385 | Response to lipid | ESR1 JAK2 MAPK8 CCND1 EGFR AR SRC | | | | | |
|  | 1.91E-06 | 9 | 7.515991 | Intracellular signal transduction | ESR1 JAK2 MAPK8 CCND1 CDK2 EGFR AR PTK2 SRC | | | | | |
|  |  |  |  |  |  |  |  |  |  |  |
|  | 5.65E-08 | 5 | 173.4855 | Mammary gland epithelium development | ESR1 JAK2 CCND1 AR SRC | | | | | |
|  | 1.06E-07 | 9 | 12.44323 | Response to oxygen-containing compound | ESR1 JAK2 MAPK8 CCND1 CDK2 EGFR AR PTK2 SRC | | | | | |
| MF | 5.23E-07 | 8 | 15.43268 | Cellular response to oxygen-containing compound | ESR1 JAK2 MAPK8 CDK2 EGFR AR PTK2 SRC | | | | | |
|  | 5.88E-07 | 5 | 83.31871 | Mammary gland development | ESR1 JAK2 CCND1 AR SRC | | | | | |
|  | 7.53E-07 | 6 | 37.52428 | Response to steroid hormone | ESR1 JAK2 CCND1 EGFR AR SRC | | | | | |
|  | 1.36E-06 | 6 | 32.96602 | Gland development | ESR1 JAK2 CCND1 EGFR AR SRC | | | | | |
|  | 1.91E-06 | 7 | 17.01557 | Positive regulation of cell population proliferation | ESR1 JAK2 CCND1 CDK2 EGFR AR PTK2 | | | | | |
|  | 1.91E-06 | 7 | 17.76575 | Response to hormone | ESR1 JAK2 CCND1 EGFR AR PTK2 SRC | | | | | |
|  | 1.91E-06 | 7 | 17.43385 | Response to lipid | ESR1 JAK2 MAPK8 CCND1 EGFR AR SRC | | | | | |
|  | 1.91E-06 | 9 | 7.515991 | Intracellular signal transduction | ESR1 JAK2 MAPK8 CCND1 CDK2 EGFR AR PTK2 SRC | | | | | |
|  |  |  |  |  |  |  |  |  |  |  |
|  | 0.001077 | 4 | 21.51073 | Focal adhesion | JAK2 EGFR PTK2 SRC | | | | | |
|  | 0.001077 | 4 | 21.19572 | Cell-substrate junction | JAK2 EGFR PTK2 SRC | | | | | |
|  | 0.001077 | 5 | 13.963 | Anchoring junction | JAK2 CCND1 EGFR PTK2 SRC | | | | | |
| CC | 0.002329 | 6 | 6.668422 | Cell junction | JAK2 MAPK8 CCND1 EGFR PTK2 SRC | | | | | |
|  | 0.002995 | 5 | 8.776469 | Synapse | JAK2 MAPK8 EGFR PTK2 SRC | | | | | |
|  | 0.003162 | 2 | 115.1313 | Cyclin-dependent protein kinase holoenzyme complex | CCND1 CDK2 | | | | | |
|  | 0.004959 | 7 | 3.923484 | Nucleoplasm | ESR1 JAK2 MAPK8 CCND1 CDK2 AR SRC | | | | | |
|  | 0.004959 | 3 | 21.10741 | Membrane raft | JAK2 EGFR SRC | | | | | |
|  | 0.004959 | 3 | 21.10741 | Membrane microdomain | JAK2 EGFR SRC | | | | | |
|  | 0.006404 | 4 | 8.674277 | Endosome | JAK2 CDK2 EGFR SRC | | | | | |
|  |  |  |  |  |  |  |  |  |  |  |
| KEGG | 4.86E-11 | 6 | 159.9719 | Endocrine resistance | ESR1 MAPK8 CCND1 EGFR PTK2 SRC | | | | | |
|  | 4.86E-11 | 8 | 38.23229 | Pathways in cancer | ESR1 JAK2 MAPK8 CCND1 CDK2 EGFR AR PTK2 | | | | | |
|  | 1.08E-09 | 5 | 180.9206 | Prolactin signaling pathway | ESR1 JAK2 MAPK8 CCND1 SRC | | | | | |
|  | 1.08E-09 | 6 | 77.14382 | Chemical carcinogenesis | ESR1 JAK2 CCND1 EGFR AR SRC | | | | | |
|  | 1.44E-07 | 5 | 63.32222 | Focal adhesion | MAPK8 CCND1 EGFR PTK2 SRC | | | | | |
|  | 1.44E-07 | 5 | 62.69527 | Proteoglycans in cancer | ESR1 CCND1 EGFR PTK2 SRC | | | | | |
|  | 4.14E-07 | 4 | 120.6138 | ErbB signaling pathway | MAPK8 EGFR PTK2 SRC | | | | | |
|  | 6.49E-07 | 4 | 104.449 | Prostate cancer | CCND1 CDK2 EGFR AR | | | | | |
|  | 1.59E-06 | 5 | 35.77527 | PI3K-Akt signalling pathway | JAK2 CCND1 CDK2 EGFR PTK2 | | | | | |
|  | 1.75E-06 | 4 | 77.34012 | FoxO signaling pathway | MAPK8 CCND1 CDK2 EGFR | | | | | |
